# Community health worker insights on promoting research engagement with diverse populations

**DOI:** 10.3389/fpubh.2022.959504

**Published:** 2023-01-11

**Authors:** Cynthia M. Killough, Annemarie Madaras, Christina Phillips, Jennifer Hettema, Venice Ceballos, Jesus E. Fuentes, Heidi Rishel Brakey, Katherine Wagner, Kimberly Page

**Affiliations:** ^1^Clinical and Translational Science Center, UNM Health Sciences Center, University of New Mexico, Albuquerque, NM, United States; ^2^Department of Family and Community Medicine, UNM Health Sciences Center, University of New Mexico, Albuquerque, NM, United States; ^3^LifeStance Health, Scottsdale, AZ, United States; ^4^Community Health Worker Initiatives, UNM Health Sciences Center, University of New Mexico, Albuquerque, NM, United States; ^5^Department of General Internal Medicine, UNM Health Sciences Center, University of New Mexico, Albuquerque, NM, United States

**Keywords:** underserved and unserved populations, community health worker (CHW), barriers to research, underrepresentation, racial minorities, ethnic minorites

## Abstract

Representation of diverse populations in health research enhances our ability to understand the factors that impact health, generalize results, implement findings, and promote social justice. The primary objective of the study was to understand the unique perspectives of frontline community health workers (CHWs) to identify actionable barriers and facilitators that may impact representation of diverse groups in health research. Focus groups with CHWs were conducted followed by thematic analysis. Results revealed five main themes: barriers/risks to research participation, facilitation of research, CHW roles, recommendations, and transparency. A novel finding was that some CHWs see themselves as both facilitators and gatekeepers. As facilitators, CHWs ensure their patient populations receive resources and benefit from being involved in research; as gatekeepers CHWs feel that they protect patient populations from experiencing further trauma, especially when engaging in research. Recognizing that in many communities there is a high reliance and trust with CHWs, can promote genuine and informed participation at all stages of research.

## Introduction

In the U.S. significant health disparities may be explained, in part, by inadequate representation of diverse racial/ethnic and other sociocultural groups in clinical research (1). Disparities in clinical research compounds other social determinants of health and widens the gap in receipt of quality health services. Representation of diverse populations in clinical and translational research enhances researchers' ability to understand the factors that impact health, generalize results, implement findings and promote social justice. Underrepresentation is also a matter of health equity in that the lack of diverse populations in clinical trials and health research reduces opportunities to finding issues that are pertinent to these groups thus reducing treatment options as well (2, 3).

Federally, efforts to include underrepresented populations were supported by the National Institutes of Health (NIH) Revitalization Act of 1993, where the NIH was directed to establish guidelines for inclusion of women and minorities in clinical research (4). Despite these mandates and the demonstrated benefits of including ethnically diverse and medically underserved populations in research, inclusion remains a struggle (5), prompting investigation into factors that might impact representation.

For racial/ethnic minority participants, there are many barriers to research participation, including logistical concerns such as childcare, schedule conflicts, lack of transportation, language barriers, and stigma of research (6). Patients who are underinsured or uninsured may have a significantly harder time participating in clinical trials requiring costs of care (7). Additionally, the U.S.' history of the exploitation of minorities and marginalized communities for research has led to mistrust, fear, and eroded confidence in not only research and researchers but also includes hospitals, institutions, medical advances, medical professions and many more (8). A widely cited example of this occurred when Havasupai Tribe members consented to give blood as part of a diabetes research study, but later learned the samples had been used for other sensitive genetic studies without their knowledge or consent (9). Another example includes the now infamous Tuskegee Syphillis Study with socially and economically marginalized Black men (8).

The inclusion of representative community members, such as Community Health Workers, in the research process, may provide a solution to address barriers to research participation. Community health workers (CHWs) are members of larger healthcare teams who provide direct services such as screening, education, and counseling, as well as supporting delivery of and promoting access to other services. CHWs go by many different titles and in New Mexico are often referred to as Promotores/Promotoras de Salud (or Promotor/a) (10) and Community Health Representatives (CHRs) (11). According to the World Health Organization, CHWs are individuals who “should be members of the communities where they work, should be selected by the communities, should be answerable to the communities for their activities…(12)*.”* As extensions of communities, CHWs may be advantaged to play important roles in the research process and their involvement may facilitate increased representation.

As members of communities served by health systems, CHWs are uniquely positioned to build trust and serve as liaisons between underrepresented community members and researchers. A review of the literature shows CHWs have played important roles in many health research projects already: CHWs have assisted in research focused on the planning, assessment, and implementation of hypertension and diabetes prevention interventions (13, 14), the delivery of child health services in rural communities (15), and the translation of research to practice in several contexts (16).

New Mexico is a geographically vast state, primarily composed of rural areas (17), the majority of people are Hispanic or Native American (18), and rates of poverty and other social determinants of health are high (19). In the current study, our investigative team conducted focus groups with a large cohort of CHWs in New Mexico to gather insights on practical strategies for promoting the inclusion of representative participants in research. In addition, CHWs are commonly found members of health teams in the state. These aspects make our state an ideal setting in which to explore factors affecting underrepresentation of diverse populations in health research. Furthermore, while inclusion of CHWs in the research process has been implemented with demonstrated success in practice, we may also be able to leverage the unique insights of this group by understanding their perspectives on research strategies that enhance or inhibit our ability to engage diverse groups in research.

## Methods

The protocol for this study was reviewed and approved by the Institutional Review Board (IRB) of the University of New Mexico (UNM) Health Sciences Center (HSC) Human Research Protections Office (#19-606).

From the inception of this study our CHW expert author (VC) has been involved and consulted for best practices and guidance- even in consideration if this study should be pursued and if it would be of interest to CHWs. Through connections and previous collaboration efforts on projects, we reached out to the Director of the Pathways to a Healthy Bernalillo County (20) (referred to as Pathways hereafter) to strategize how best to recruit CHWs from all areas in the city. Pathways “…uses a version of the Pathways Model to identify vulnerable, underserved residents and connect them to health and social services. Clients are identified through interagency referral among the program's network of 13 community-based organizations. Community health [workers] help clients access additional health and social services, assist with coordination of care, and monitor client progress” (20). The Pathways Director invited researchers to come to a Pathways meeting to promote this research study. CHWs working with various organizations (including the UNM HSC as well as other community organizations, clinics, and agencies throughout Albuquerque) regularly attend, and this opportunity provided researchers an optimal chance to a diverse group of CHWs for the study. The Pathways Director noted that many if not all of the CHWs had previous experience with research through various projects.

The study took place in January of 2020 in a community facility. Before the focus groups began we distributed a written IRB-approved information sheet, provided a verbal overview of the study, and also distributed an anonymous survey with basic demographic information. Forty-two CHWs were invited to participate in the study, and all (42) accepted. Participants were divided into five (*N* = 5) focus groups, with 8 CHWs in 3 groups, and 9 CHWs in the other two groups. The groups were well spaced out within a very large conference room with a trained facilitator at each group. One focus group was conducted in Spanish by a fluent facilitator and participants self-selected into that group. We designed a semi-structured focus group guide to gain actionable perspectives to help researchers increase representation of underserved communities in their projects. Prompted by the interview guide, facilitators also allowed the CHWs to lead the focus group conversations in the direction they wanted with the understanding that health research was the focus. Following are the primary questions from the guide that were asked of each group:

- What do you think about when you hear the word “research”?
- What stigma is associated with research in your communities?
- If you were part of a research study, what would you expect from the researchers during and after the study is over?
- What strategies should researchers use and avoid when approaching clinics, providers, or patients about research?
- What strategies should researchers avoid when approaching clinics, providers, or patients about research implementation/dissemination?

Each group discussion was audio recorded and transcribed verbatim. Each participant received a $40 merchandise card.

### Analysis strategies

Transcripts of the audio recordings, including English and Spanish, were done by a professional transcription company. The coding team (CK, AM, CP, JH, and JF) analyzed transcripts using Consensual Qualitative Research techniques (21) and Vaismoradi et al.'s (22) stages to thematic development in qualitative content coders reviewed transcripts individually, came together to identify broad themes and underlying subthemes, assigned participant quotes to those subthemes, and finalized the themes. Two Spanish-fluent analysts (CK and JF), analyzed the Spanish speaking transcript, translated them back into English and then integrated those participant quotes into the larger analysis.

## Results

All 42 participants were asked to complete a short anonymous survey including demographics, gender, age, race/ethnicity, and work setting. Responses were submitted by 33 (79%) of the participants at the end of the focus groups. Of these 33, 76% were female, 36% were between the ages of 35–44 years, 24% identified as Latina/o, 42% were not certified CHWs, and 54% reported working in social services organizations (see Table 1).

**Table 1:** Community health worker demographic information.

	***n* = 33**
**Gender**	
Female	25 (76%)
Male	8 (24%)
**Age**	
18–24 years	2 (6%)
25–34 years	6 (18%)
35–44 years	12 (36%)
45–54 years	8 (24%)
55–64 years	5 (15%)
**Ethnicity (select all that apply)**	
White	3 (9%)
Hispanic white	6 (18%)
Hispanic non-white	4 (12%)
Latino	8 (24%)
Native American	2 (6%)
Alaskan native	0
Native Hawaiian	0
Pacific Islander	2 (6%)
African American	3 (9%)
Asian	5 (9%)
Other	4 (12%)
**Certified community health worker? (*****n*** = **32)**	
Yes	11 (33%)
No	14 (42%)
Working toward becoming certified	7 (21%)
**Work setting (select all that apply)**	
Hospital	3 (9%)
Clinic (not hospital setting)	9 (27%)
Social service organizations	18 (54%)
Academic setting	0
Community mental health center	4 (12%)
Primary care	1 (3)
Correctional facilities	0
Homeless shelter(s)	6 (18%)
City/County department(s)	1 (3%)
Public school	2 (6%)

Excel was used for the coding and thematic analysis. Five broad themes were identified by the coding team: (1) *Barriers/Risks* that may deter individuals from participating in research or deter CHWs from promoting research participation, (2) *Facilitation of research* such as factors to increase participation in research or encourage CHWs to facilitate research involvement, (3) *CHW roles* and the ways those roles impact decision making about research and their impact on research participation, (4) *Transparency* that CHWs requested of researchers and a desire that any and all information about the research project be provided up front, and (5) *Recommendations* or concrete tips, advice, and strategies CHWs recommended for researchers seeking to work with their populations (see Table 2). Below, we provide a description of each identified theme.

**Table 2:** Codebook and quote examples from Community Health Worker (CHW) focus groups.

**Theme**	**Description of theme**	**Exemplar quote**
Barriers/risks	Any mention of barriers to research such as past experiences, quality improvement, special populations. considerations, stigma, etc.	
*Subtheme 1. Special populations barrier/risks*	CHWs describe barriers or risks that specific special populations, such as underserved or underrepresented populations, face in order to participate in research.	“If the research is going to get the name [of the participant] and the nationality or immigrant status…some people with problems with immigration don't want to provide any [identifying information].”
*Subtheme 2. Nothing is done with the results*	CHWs describe any barriers or challenges to research due to the uncertainty the result will have a positive impact or given back to their community.	“Are you going to come back and give the report back to our community? Because if [community members are] participating, they want to know why. It's because sometimes they say, ‘Well, the community doesn't want to participate.’ Yes. We don't want to participate because you never come back and tell me the results.”
*Subtheme 3. Research is not for minorities*	CHWs describe perceptions that people of color do not think research is for them and that societal racism extends into research.	“The stigma…is that Black people don't matter, so they feel like they don't need to do research on us because there's not that many of us in Albuquerque let alone the state of New Mexico. So, when they do research, they don't even include us in it.”
Facilitation of research	CHWs describe how they perceive research as valuable particularly when it directly informs the patient population they serve.	“You get to see what populations need more help, need more resources…so that we can try to fix that.”
Community Health Worker Roles	The roles that CHWs fulfill within their patient/client interactions and meanings they attach to them.	
*Subtheme 1. Accountable to patients*	Any mention of the CHWs expressing that they are ensuring their patients are informed of research outcomes from the studies patients have participated in.	“…Whenever we're working with the researcher… there should be a date set… When are you going to come back and report back to the community? So we can let our community know, by this date…we're going to get the research back. We're going to get the report, whether it's good or whether it's bad.”
*Subtheme 2. Gatekeepers*	CHWs describe how they must weigh the risks and benefits of their patients' participation in research studies.	“They're somebody you've been seeing for a while. You do care about them. You want to know that there is nothing bad going to happen. I mean, that they are safe if they are going to be answering or doing whatever it is…through your research. That they're going to be safe.”
Transparency	References made to researchers needing to be up front and clear in all aspects of the research process.	“Pienso que deben clarificar bien el concepto de lo que quieren ustedes investigar y aclarárselo bien a la comunidad, a nivel, porque no es por la falta de educación ni nada. Entonces, no conocen nada sobre lo que es… una investigación.” (I think that you should clarify well the concept of what you want to investigate and clarify it well to the community, at the level, because it is not due to the lack of education or anything. So, they don't know anything about what an investigation… is.)
Recommendations	CHWs discuss recommendations for researchers to approach and engage with their populations.	
*Subtheme 1: Remuneration or resource transaction*	CHWs describe ways researchers can think of ways, monetarily or by providing other resources, to pay their populations for their time and contributions.	“We usually have to have a snack or a daily bus pass for them to even consider going to a focus group or something. So that would be something… we would have to think of ahead of time or else they're not going to participate.”
*Subtheme 2: Ensure a physical presence*	Any mention to researchers needing to approach and engage with patient populations in-person.	“Showing up a couple days [in advance] that way we get familiar with your face…It's such an interpersonal job anyway. The position of an aggregator or CHW, you become part of a family unit with your clinics and the clinical staff or whoever else you're working with…we get wrapped into the whole tapestry. It's personal.”
*Subtheme 3: Work within the existing system*	Any mention to researchers looking into systems in place surrounding their populations and the professional hierarchies established within organizations or agencies in order to overcome barriers to research.	“Contact a leader in the community and then the leader explains to the community because…if they respect someone in the community, they're going to hear everything [about the research] and participate.”
*Subtheme 4: Words matter*	Any mention of researchers needing to be aware of the language they use and avoiding certain words or phrases around CHW's patient populations.	“Avoid words like alien, illegal. Even when we're talking about substance use, I think saying substance abuse, drug abuse, has a certain connotation and when you're talking to users.”

### Theme 1: Barriers/Risks

We coded participant statements as *Barriers/Risks* if CHWs identified challenges to research participation. Three subthemes emerged: special populations barriers/risks, nothing is done with the results, and research is not for minorities.

*Subtheme 1: Special populations barriers/risks*. CHWs discussed barriers or risks that specific special populations, such as underserved or underrepresented populations, face in order to participate in research. One CHW mentioned “If the research is going to get the name [of the participant] and the nationality or immigrant status…some people with problems with immigration don't want to provide any [identifying information].” Another CHW also brought up concerns regarding legal risks of research participation, “I think with the current administration [2016–2020], there's a lot of people because of their immigration, they are scared to come out to participate in any research because they are not sure what is the purpose of the research or that kind of things,” and another CHW continued,

“…We serve a lot of people that don't have a lot of resources…it's a pretty negative connotation especially when we're talking about immigrant communities with legal ramifications. Why am I going to involve myself in something that includes my information, my opinion, when I don't really know what's going to happen or I don't know if I'm going to be able to stay safe?”

*Subtheme 2: Nothing is done with the results*. An issue that CHWs brought up was that participants may not see the value in participating because they are not confident that results and the findings will positively impact or be given back to their community as one CHW pointed out: “Are you going to come back and give the report back to our community? Because if [community members are] participating, they want to know why. It's because sometimes they say, “Well, the community doesn't want to participate.” Yes. We don't want to participate because you never come back and tell me the results.” Another CHW added, “That's the other issue. We do research. We find the problem. And we give [researchers] ideas of solutions. But then nothing happens. And then the organizations are not held accountable.”

*Subtheme 3: Research is not for minorities*. CHWs discussed perceptions that people of color do not think research is for them and that societal racism extends into research. “The stigma…is that Black people don't matter, so they feel like they don't need to do research on us because there's not that many of us in Albuquerque let alone the state of New Mexico. So, when they do research, they don't even include us in it.” Another CHW added, “Well, we have the same situation… Latinos. Most of the research programs are for other kind of people but no Latinos, no African American, no different people.”

### Theme 2: Facilitation of research

CHWs indicated they perceive research as valuable and that it can improve patient care and outcomes, particularly when it directly informs the patient population they serve: “We can't just take up every research [project] that comes up…it has to be important.” Another CHW stated, “You get to see what populations need more help, need more resources…so that we can try to fix that.” In one case, a CHW highlighted that research actually was important in supporting the value of CHWs within clinics. CHWs also reported valuing monetary benefits that are paid directly to research participants as well as funds that might be provided to a clinic or program to support as part of research collaborations.

### Theme 3: CHW roles

Two subthemes emerged under CHW Roles. First, CHWs discussed the importance of the community trust they hold, and the responsibility to be accountable to their patients, which extends to ensuring their patients are informed of research outcomes from the studies patients have participated in. For example, one person suggested, “…Whenever we're working with the researcher… there should be a date set… When are you going to come back and report back to the community? So we can let our community know, by this date…we're going to get the research back. We're going to get the report, whether it's good or whether it's bad.” A second subtheme was that some CHWs see themselves as gatekeepers between researchers and the community they serve, and they must weigh the risks and benefits of their patients' participation in studies. CHWs expressed the imperative priority of protecting vulnerable patient groups from experiencing unforeseen risks of research participation, such as those who have histories with trauma, or who are undocumented: “They're somebody you've been seeing for a while. You do care about them. You want to know that there is nothing bad going to happen. I mean, that they are safe if they are going to be answering or doing whatever it is…through your research. That they're going to be safe.”

### Theme 4: Transparency

“Transparency” emerged as the need for researchers to be up front and clear in all aspects of the research process and to be able to communicate these pieces effectively to participants. One Spanish speaking CHW said,

“Pienso que deben clarificar bien el concepto de lo que quieren ustedes investigar y aclarárselo bien a la comunidad, a nivel, porque no es por la falta de educación ni nada. Entonces, no conocen nada sobre lo que es… una investigación.” (I think that you should clarify well the concept of what you want to investigate and clarify it well to the community, at the level, because it is not due to the lack of education or anything. So, they don't know anything about what an investigation… is.)

Another CHW followed up: “… Every research is important to get whatever it is that the research needs or money or funding, whatever it is. But I think it's just how you ask the questions, how you do the research. What is it for? So that the people can participate honestly on the research.”

### Theme 5: Recommendations

CHWs discussed several recommendations for researchers to approach and engage with their populations. There were four subthemes that emerged: remuneration or resource transaction, ensure a physical presence, work within the existing system, and words matter.

*Subtheme 1: Remuneration or resource transaction*. CHWs recommended researchers think of ways, monetarily or by providing other resources, to pay their populations for their time and contributions, monetarily or with other resources. One CHW noted, “We usually have to have a snack or a daily bus pass for them to even consider going to a focus group or something. So that would be something… we would have to think of ahead of time or else they're not going to participate.” Another CHW added, “The patients are struggling with transportation and babysitting or things like that… what could you do to help them in order to help you?”

*Subtheme 2: Ensure a physical presence*. Another recommendation was researchers need to approach and engage with their populations in-person as one CHW discussed:

“Showing up a couple days [in advance] that way we get familiar with your face…It's such an interpersonal job anyway. The position of an aggregator or CHW, you become part of a family unit with your clinics and the clinical staff or whoever else you're working with…we get wrapped into the whole tapestry. It's personal.”

Another CHW recommended,

“I think coming in with a physical brochure and… hand[ing] out the brochures, meeting with the supervisor…. And getting their thoughts on the approach and the clientele that they work with. And that way, when they do come for the study [patients] know… what to expect or what kind of reaction they may be getting. It prepares the researcher as well. Kind of doing homework.”

*Subtheme 3: Work within the existing system*. CHWs recommended researchers look at the systems in place surrounding their populations and the professional hierarchies established within organizations or agencies for which they wish to engage. For example, one CHW recommended researchers could “Contact a leader in the community and then the leader explains to the community because…if they respect someone in the community, they're going to hear everything [about the research] and participate.” Another CHW added,

“Well, I think that you have to approach the director or whoever's in the top before us. After that… What is the research all about? And then go from there. I mean, we're at the end. We're at the communication between you guys, the research, and the family because we have the connections, because we work with the families, but it just depends on what the study is all about and then us, we try to do our best.”

*Subtheme 4: Words matter*. The final recommendation that emerged was that the words researchers use when addressing participants, even when bringing up the topic of research, mattered. For example, CHWs recommended researchers, “Avoid words like alien, illegal. Even when we're talking about substance use, I think saying substance abuse, drug abuse, has a certain connotation and when you're talking to users.” Another CHW agreed: “Alien, for example, it's written and it's the proper word because the politics. The government put it. But in reality, it's not so friendly because I'm not Hispanic. I'm not an alien.” A third CHW said, “But you see also, the wording, if you go to a community that is burned out for so-called being the instrument of research, don't come and say, “I'm going to do a research.” I would remove completely the *research* word.”

While CHWs are often called upon to facilitate aspects of research as well as observe the research process within health settings and hear patient perspectives (23, 24), we interpreted the emerging themes to mean that some CHWs see themselves as both facilitators and gatekeepers to their patient populations. As facilitators, CHWs ensure their patient populations get the resources they need and benefit from being involved in research. As gatekeepers, CHWs feel they must protect patient populations from experiencing more trauma, including when engaging in research. Both of these roles were encompassed in a back-and-forth discussion by the CHWs, “…So being that we're all community health workers, shouldn't it be our responsibility, if we're really good at doing our job, to piggy-back off of the researchers and find out, “Hey, what's going on? What was done with that study?” and then we go back and relay it to the community?,” to which their colleague responded “…I am accountable to the community… we're accountable to the community.”

## Conclusion

The primary objective of the current study was to understand the unique, frontline perspectives of CHWs and identify actionable barriers and facilitators that may impact representation of diverse groups in research. A novel dichotomy emerged in the way CHWs view their own roles that, to our knowledge, has not been previously published. Some CHWs see themselves as both facilitators and gatekeepers to their patient populations. CHWs are integral to bringing a viewpoint to their patients. Researchers may not be aware that for some communities CHWs represent a group with whom there is a large power imbalance. Recognizing that in many communities there is a high reliance and trust with CHWs, can promote genuine and informed participation at all stages of research. CHWs emphasized the need for researchers to be transparent from the beginning of the project and communicate all aspects of the research process in a way that is effective and understandable by all participants. Furthermore, CHWs reported they feel research is not for minorities or people of color because they are often only a fraction of the population at large (i.e., only 2.6% of NM is Black or African American) (25). This is alarming if the goal is to increase representation from racial/ethnically diverse groups in health research.

It is important to note that a few of the themes that emerged from the focus groups are in line with previous studies. Under the *Special population barriers/risks* subtheme, CHWs discussed participant's immigration status and the challenges facing these populations in research participation. Undocumented immigration status impedes many individuals from participating in many different facets of health research and healthcare which is a largely discussed prevailing issue among researchers and healthcare providers (26–28). The *Remuneration or resource transaction* subtheme is also documented in the literature; however, opinions on monetary compensation and context are still debated. Still, in accordance with the recommendations of the CHWs in our study, it is advised to consider compensation for research participation. Furthermore, the *Nothing is done with the results* subtheme aligned with our own goals of data dissemination and knowledge of best practices, and as this paper went into submission a date and time was set to disseminate the results of this study back to the CHWs at Pathways.

Considering these themes, there are significant opportunities to continue and expand this knowledge and promote research engagement with historically excluded diverse populations. First, working with CHWs from the inception of a study could not only inform the research itself but could also provide a unique opportunity to strategize ways in which participants and the community could benefit from the study (i.e., the study could be tied to a community education effort or helping to secure sustainable funding for CHWs). Next, when researchers request assistance from CHWs, it is worthwhile to consider the multifaceted nature of how CHWs view their roles and expertise. Also, less time is needed focusing on convincing CHWs the value of research and focus could instead be placed on collaboration to highlight how research can support their work efforts and their patients. In addition, from the start of the project, researchers could find ways to help CHWs better understand participant confidentiality and how their patient's contact information will be stored and used. Next, researchers could take time to show CHWs how their patients can benefit from research, what incentives and resources are available, and how they plan on disseminating findings once the project has ended. Finally, researchers could prioritize dissemination of their results back to communities in accessible and meaningful ways, using plain language. This is consistent with other research showing dissemination of findings encourages future participation in research and fosters trust between researchers and participants (24). In reference to Theme 1: Barriers/Risks and its subsequent subthemes it appears that a common conception about research among CHWs and their patient populations is that research does not leave anything of value for the participants and furthermore does not contribute to the community; arranging ways in which researchers could bring the data back to the participants and the community early in the study could help in changing these negative perceptions of research and researchers. We would also like to note that at the time of submitting this paper, we have been in contact with Pathways to set up a time where the results of this study can be disseminated back to CHWs with recommendations on how best to do so (i.e., power point presentation, infographic, etc.).

The current study also provided opportunities for reflexivity on the researcher's experiences and training and how this affected the way the data was collected and interpreted. Our research team worked in varying departments across our university campus, however we all fall under the community engagement umbrella in health research. Our team is made up of qualitative experts- some who have prior experience working directly with CHWs and others were new to the field. The qualitative method we used allowed us to view CHWs as experts and we followed their lead through the interview guide. While analyzing the data a strength of our team is the varying degree to which we had experience working with CHWs which allowed us to view the data more objectively as the team discussed various themes that emerged. We also acknowledged that however objective we tried to be, we were still not CHWs which is why we were grateful for the insight and assistance from our collaborative CHW expert coauthor (VC). We also understood that while one person is not representative of all CHW voices, their experience and expertise was still valued as a guide for our efforts.

Several limitations exist in the current study. We were unaware of the need for a focus group to be led by a Spanish speaking facilitator until we got to the CHW meeting. Fortunately, one of our researchers was fluent and could facilitate a group in Spanish but had to translate the questions in the moment. Additionally, the focus groups were held simultaneously in the same large open space, rather than separate, private spaces. As such, there was crosstalk and lack of privacy that may have prevented some participants from sharing more. We did have some missing data on participant demographics, as not all the participants returned the demographic survey, which did include instructions saying they were not obligated to finish or take the survey. The findings of the current study are also from CHWs who serve a majority Hispanic population. These factors (missing data and CHW population focus) may limit generalizability of our results. Finally, the COVID-19 pandemic began soon after the focus groups and disrupted workflow. During this time researchers were unable to meet as they adapted to stay-at-home orders and other remote work guidelines.

Moving forward there are various actions that can be done to assist both CHWs and researchers to increase patient research participation. For example, CHW certification programs could look into ways of incorporating research education into their curriculum. This is in line with recommendations from the CHW Core Consensus (C3) project which included *participating in research* within CHW's scope of practice (29). CHWs who understand research processes and research in general can better aid their patients who may be interested in research participation. As was made clear by the CHWs in the current study, the purpose of research is not always clear, meaning more information is needed on the front-end of projects before individuals decide to participate. Also, exposing CHWs to other research approaches (e.g., Rationale for Research Participation Framework) could bolster their knowledge about why participants choose to participate in health research, including risk-benefit and reciprocity perspectives (30). There is considerable room for improvement from researchers and institutions, considering some barriers to engaging diverse populations are well documented and restated in the current study. Finally, opportunities such as taking Community-Based Participatory Research training (31) may help researchers be more responsive to community needs and design their studies in a more community-based and community-friendly manner.

## Data availability statement

The raw data supporting the conclusions of this article will be made available by the authors, without undue reservation.

## Ethics statement

The studies involving human participants were reviewed and approved by University of New Mexico Health Sciences Center Human Research Protections Office (#19-606). Written informed consent for participation was not required for this study in accordance with the national legislation and the institutional requirements.

## Author contributions

CK, AM, CP, JH, and JF contributed to the analysis and writing on the first draft and subsequent versions thereafter. VC, HR, and KW assisted with subsequent versions of the manuscript. KP consulted on the study itself and provided guidance and support throughout the writing process. All authors contributed to this paper and fulfill the authorship criteria and reviewed and approved the final draft.
